# Supplementary material for: Utilization of Ureteral Access Sheath in Retrograde Intrarenal Surgery: A Systematic Review and Meta-Analysis
Source: Medicina (Kaunas). 2024 Jul 2;60(7):1084. doi: 10.3390/medicina60071084 (PMC11278831; doi:10.3390/medicina60071084)

Supplementary Material

Table S1. Characteristics of the included studies.

Author	Year	Group	Procedures, n	Age, years	Stone burden, mm	Multiple Stones (2 ≦), n (%)	Preoperative stent, procedures, n (%)	Postoperative stent, procedures, n (%)	Ureteroscopy	Definition of SFR	The decision of UAS usage
Singh	2023	UAS	41	38.95±12.15	14.71±4.57	NR	100%	100%	F	Residual stone ≦ 3mm	Randomized
		Non-UAS	40	39.13±11.34	15.32±4.97				F		
Ozimek	2022	UAS	98	54 (38; 68)[a]	7 (5; 10)[a]	71 (72.45%)	96 (97.96%)	98 (100.00%)	S+F:F = 63:35	NR	Surgeon's preference
		Non-UAS	185	58 (45; 68)[a]	6 (4; 9)[a]	76 (41.08%)	172 (92.97%)	173 (93.51%)	S+F:F = 135:50		
Ecer	2022	UAS	40	47.95±15.2 and 46.35±14.1	14.65±5.6 and 12.65±3.6	6 (30%) and 6 (30%)	0 (0%)	20 (100%) and 19 (95%)	F	Residual stone ≦ 3mm	Randomized
		Non-UAS	20	50.5±12.4	14.95±6.5	5 (25%)	0 (0%)	19 (95%)			
Cristallo	2022	UAS	43	54.1±14.7	11.5±4.6	23 (53.5%)	29 (67.4%)	24 (55.8%)	F	NR	Arbitrarily
		Non-UAS	198	53.2±14.5	9.2±4	86 (43.4%)	99 (50%)	86 (43.4%)			
Bozzini	2021	UAS	92	51.4±22.1	15.82±4.12	0 (0%)	0 (0%)	92 (100%)	F	Residual stone ≦ 3mm	Randomized
		Non-UAS	89	48.3±24.8	14.11±4.89	0 (0%)	0 (0%)	89 (100%)			
Yitgin	2021	UAS	51	44.9±15.1 (20-73)	1273±1036 (251-3635)[c]	NR	NR	51 (100%)	F+S	Residual stone ≦ 2mm	NR
		Non-UAS	62	46.1±13 (20-74)	1135±1268 (73-7491)[c]			62 (100%)			
Damar	2021	UAS	30	49.58±13.8	76.59±37.47[b]	3 (10%)	0 (0%)	25 (83.33%)	F	Residual stone ≦ 3mm	NR
		Non-UAS	30	48.36±15.62	62.19±33.12[b]	1 (3.33%)	0 (0%)	22 (73.33%)			
Meier	2021	UAS	1969	58.81±15.3	6.79±4.5	NR	1079 (33.2%)	2042 (62.7%)	NR		NR

		Non-UAS	3260	54.30±16.1	8.79±5.3		942 (47.9%)	1739 (88.5%)		Completely clean	
Cooper	2020	UAS	1039	51.0±14.8	12.5±8.6	453/1330 (34.1%)	463 (34.8%)	1205 (90.5%)	S:F:S+F = 199:972:161	NR	NR
		Non-UAS	293	47.9±15.5	7.3±4.9						
Lima	2020	UAS	203	55.0±20.3	16.5±10.8	2.17±1.99[d]	56 (27.6%)	164 (82.4%)	F+S	Residual stone ≦ 2mm	Surgeon's preference
		Non-UAS	135	58.1±17.8	11.37±8.08	1.66±1.50[d]	38 (28.3%)	107 (81.1%)			
Sari	2020	UAS	1489	46.2±13.8	15.6±7.9	294 (19.7%)	72 (4.8%)	1313 (88.2%)	F+S	Residual stone ≦ 3mm	NR
		Non-UAS	319	44.9±13.5	12.53±5.9	48 (15.0%)	40 (12.5%)	201 (63.0%)			
Karaaslan	2019	UAS	81	48.8±12.1	14.9±5.7	NR	NR	NR	R+F	NR	Surgeon's preference
		Non-UAS	48		15.8±6						
Özkaya	2019	UAS	70	37.01 (16-80)	9.77	6 (9%)	NR	67 (96%)	F+S	Residual stone ≦ 3mm	Randomized
		Non-UAS	61	45.01 (19-76)	9.04	5 (8%)		60 (98.3%)			
Lildal	2018	UAS	88	55 (18-97)	574 (437-710)[e]	NR	44 (50.0%)	84 (95%)	F	NR	Surgeon's preference
		Non-UAS	92	50 (18-81)	461 (291-631)[e]		34 (37.0%)	82 (89%)			
Geraghty	2016	UAS	40	54 (7-84)	29.2 (20-60)	23/43 (53.5%)	15	64 (94.1%)	F	Residual stone ≦ 2mm	Surgeon's preference
		Non-UAS	28				11				
Traxer	2015	UAS	1494	51.2±14.98	108.3 ±114.4[b]	458/1449 (31.6%)	511	1352	F	Residual stone ≦ 1mm	NR
		Non-UAS	745	50.2±14.95	99.2±100.5[b]	194/722 (26.4%)	278	611			
Berquet	2014	UAS	157	50±15.2	15.15±9.8	1.92±1.54	39 (24%)	134 (85%)	F	Residual stone ≦ 3mm	Surgeon's preference
		Non-UAS	123	52±17.3	13.75±8.0	1.61±1.14	62 (50%)	94 (76%)			
Wang	2011	UAS	40	13.6±4.2 (4.0-20.9)	12.5±9.7 (3.0-54.0)	NR	12	38	NR	NR	NR

		Non-UAS	56	12.7±4.6 (1.5-19.9)	7.6±4.5 (0.8-27.0)		14	37			
Pardalidis	2006	UAS	48	48.5 (18±73)	7.1	NR	NR	100%	F	Completely clean	Randomized
		Non-UAS	50		7.8						
L'esperance	2005	UAS	173	49	8.7	NR	NR	77%	F	Completely clean	NR
		Non-UAS	83	47	7.3						
De Sio	2004	UAS	12	54 (26± 71) and 61 (54±68)	1.4 (1±2.5) and 0.7 (0.4±0.9)	NR	NR	NR	S	NR	NR
		Non-UAS	16	45 (18±74) and 63 (61±75)	1.6 (1.1±2.8) and 0.9 (0.4±1.2)						
Kourambas	2001	UAS	30	43.8 (21±85)	13.00	NR	NR	15	F:S=25:5	NR	Randomized
		Non-UAS	32		10.35			19	F:S=23:9		

NR, not reported; SFR, stone free rate; UAS, ureteral access sheath; non-UAS, without an ureteral access sheath; F, flexible ureteroscopy; S, semirigid ureteroscopy; R, rigid ureteroscopy; SD, standard deviation; Fr, French.
Values are given as mean ± SD (range).
[a]Given as median (interquartile range).
[b]Calculated as the sum of all stone sizes (length × width × 0.25 × 3.14159).
[c]Stone volume given as mm^3.
[d]Given as mean number of stones.
[e]Calculated volume using the formula for a sphere, given as mean (95% CI), mm^3.

Figure S1. Funnel plot of SFR.

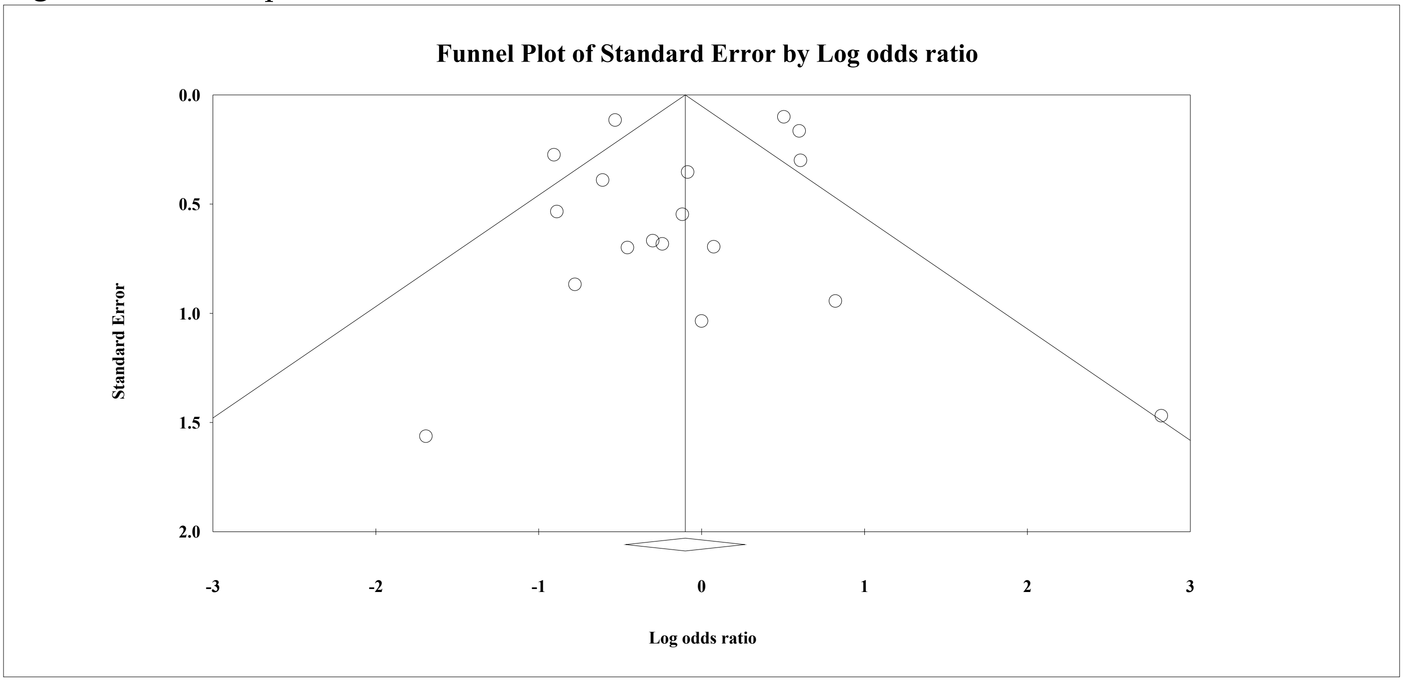

Figure S2. Funnel plot of intraoperative complications.

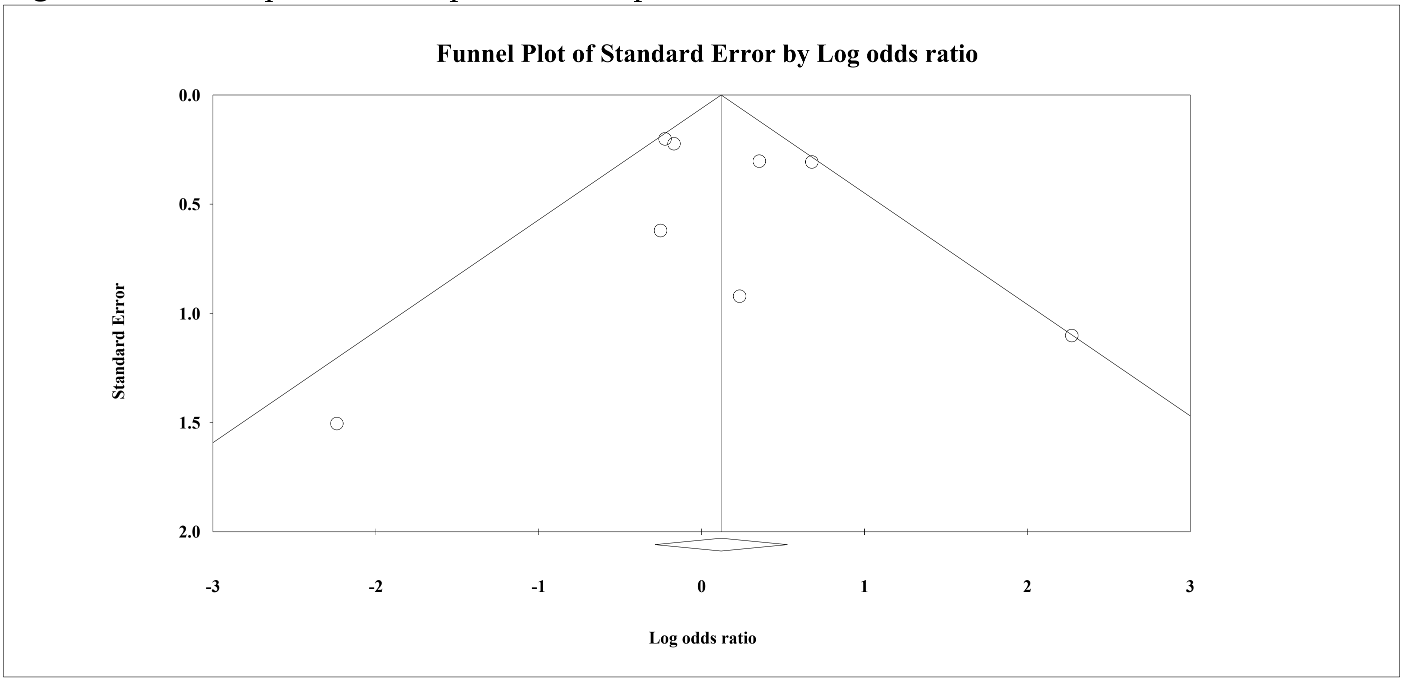

Figure S3. Funnel plot of postoperative complications.

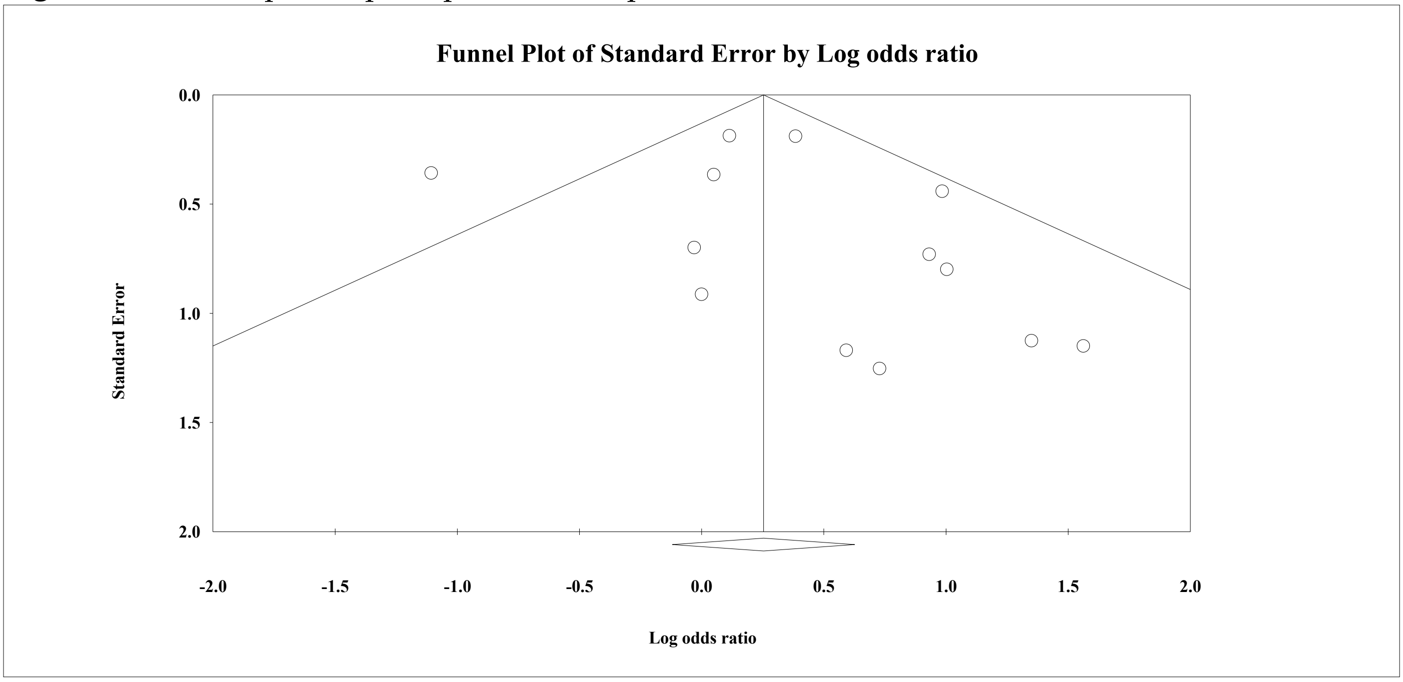

Figure S4. Funnel plot of operation time.

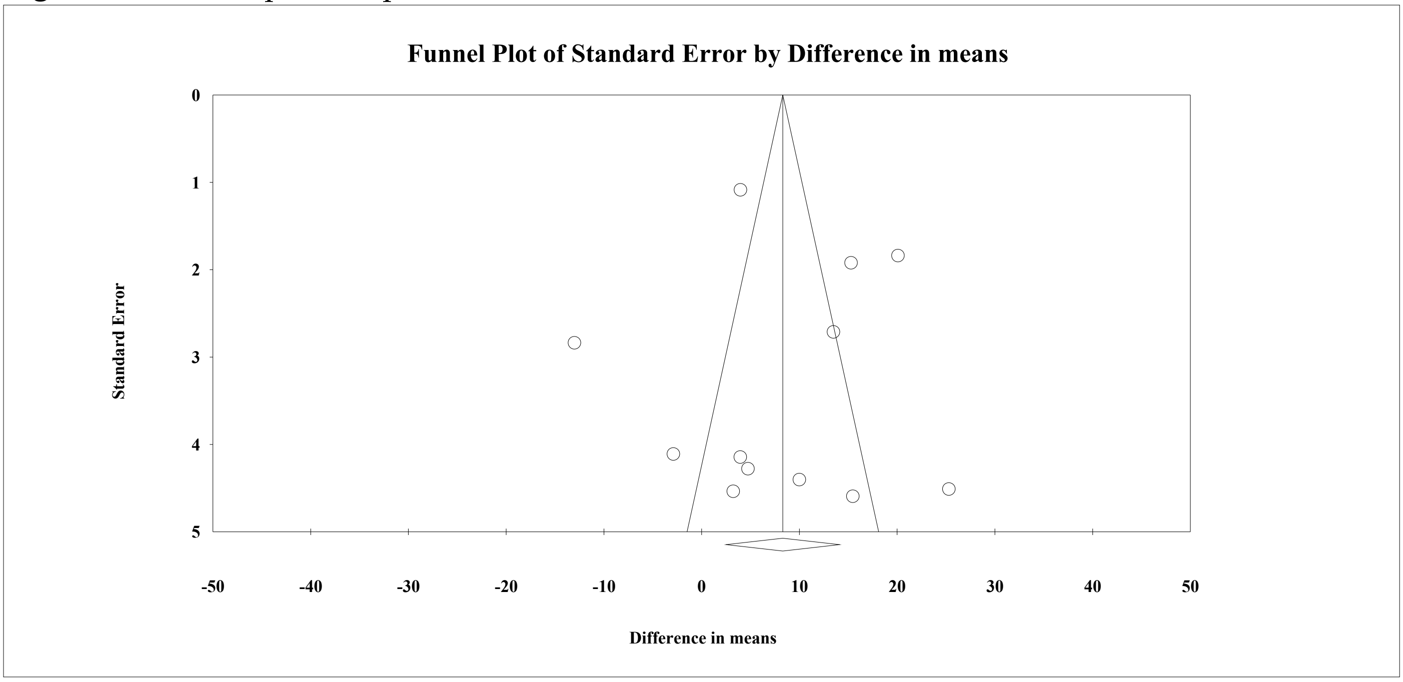

Figure S5. Funnel plot of hospitalization time.

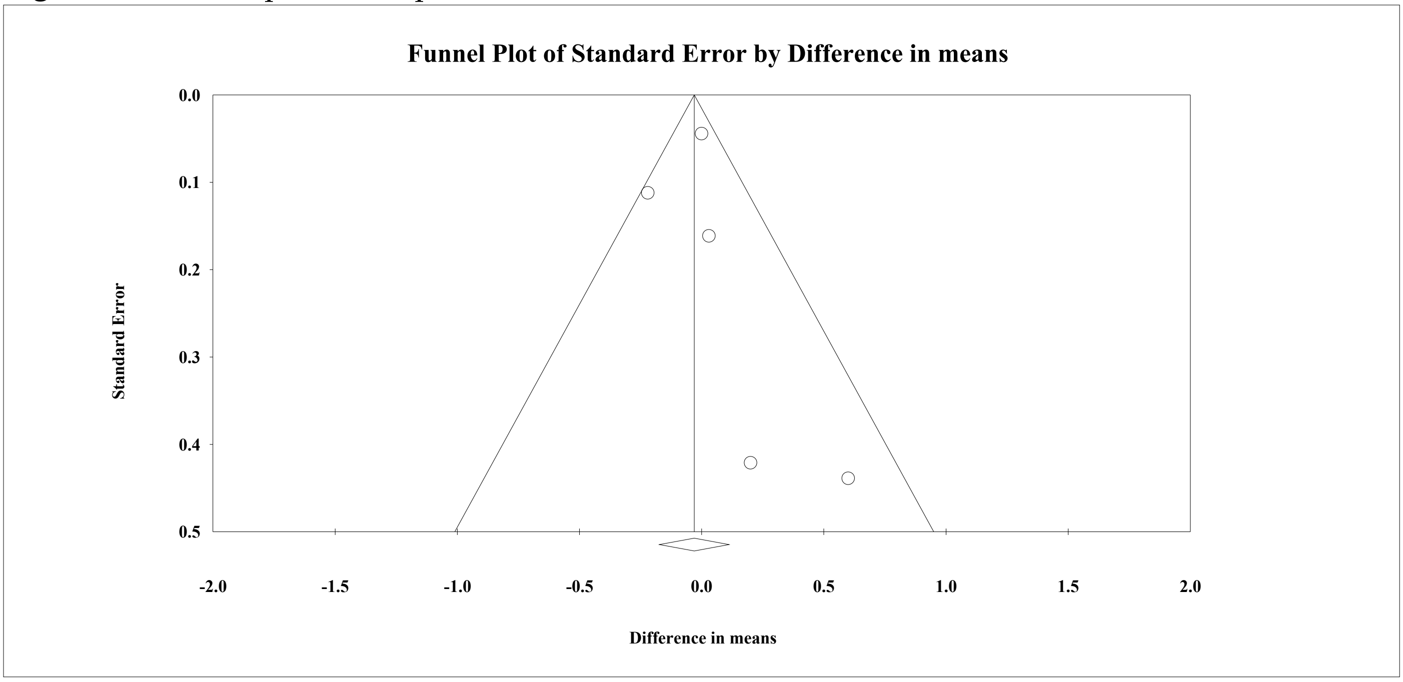

Supplement: Supplementary file 1 [file medicina-60-01084-s001.zip › Supplementary Material.pdf]
